# From Omics to Multi-Omics Approaches for In-Depth Analysis of the Molecular Mechanisms of Prostate Cancer

**DOI:** 10.3390/ijms23116281

**Published:** 2022-06-03

**Authors:** Ekaterina Nevedomskaya, Bernard Haendler

**Affiliations:** Research and Early Development, Pharmaceuticals, Bayer AG, Müllerstr. 178, 13353 Berlin, Germany; ekaterina.nevedomskaya@bayer.com

**Keywords:** omics, multi-omics, prostate cancer, androgen receptor, stratification

## Abstract

Cancer arises following alterations at different cellular levels, including genetic and epigenetic modifications, transcription and translation dysregulation, as well as metabolic variations. High-throughput omics technologies that allow one to identify and quantify processes involved in these changes are now available and have been instrumental in generating a wealth of steadily increasing data from patient tumors, liquid biopsies, and from tumor models. Extensive investigation and integration of these data have led to new biological insights into the origin and development of multiple cancer types and helped to unravel the molecular networks underlying this complex pathology. The comprehensive and quantitative analysis of a molecule class in a biological sample is named omics and large-scale omics studies addressing different prostate cancer stages have been performed in recent years. Prostate tumors represent the second leading cancer type and a prevalent cause of cancer death in men worldwide. It is a very heterogenous disease so that evaluating inter- and intra-tumor differences will be essential for a precise insight into disease development and plasticity, but also for the development of personalized therapies. There is ample evidence for the key role of the androgen receptor, a steroid hormone-activated transcription factor, in driving early and late stages of the disease, and this led to the development and approval of drugs addressing diverse targets along this pathway. Early genomic and transcriptomic studies have allowed one to determine the genes involved in prostate cancer and regulated by androgen signaling or other tumor-relevant signaling pathways. More recently, they have been supplemented by epigenomic, cistromic, proteomic and metabolomic analyses, thus, increasing our knowledge on the intricate mechanisms involved, the various levels of regulation and their interplay. The comprehensive investigation of these omics approaches and their integration into multi-omics analyses have led to a much deeper understanding of the molecular pathways involved in prostate cancer progression, and in response and resistance to therapies. This brings the hope that novel vulnerabilities will be identified, that existing therapies will be more beneficial by targeting the patient population likely to respond best, and that bespoke treatments with increased efficacy will be available soon.

## 1. Introduction

Prostate cancer is the second most frequently diagnosed tumor and sixth leading cause of cancer death in men worldwide. In 2020, an estimated 1,414,300 new cases and 375,300 deaths were reported globally [1]. These figures have risen over recent years, due, in part, to the overall growth and ageing of the population, but there is a trend towards stabilization of fatalities and even regression in some countries, probably connected to expanded screening and improved medications [2].

Prostate cancer can be diagnosed at various degrees of aggressiveness [3] and often appears as somatically independent tumor foci [4,5]. Three main development stages, namely intraepithelial neoplasia (PIN), hormone-sensitive prostate cancer (HSPC) and castration-resistant prostate cancer (CRPC), have been defined and additional subtypes proposed, chiefly based on genomic and transcriptomic profiles [3,6,7,8]. Metastases can be observed at the HSPC and CRPC stages, and they are usually clonally related in a given patient and spreading may occur between metastatic sites [9,10,11,12]. The increasing use of potent AR-targeting therapies has led to the emergence of novel CRPC subtypes, including the amphicrine, androgen receptor (AR)-low, double-negative and small-cell or neuroendocrine phenotypes [13]. Recognized risk factors for prostate cancer are age, ethnicity and environmental factors [14]. Around 5–15% of cases are attributed to hereditary factors and numerous susceptibility loci, and mutations, often in DNA repair genes, have been linked to them [14].

The driving role of AR signaling in early and late prostate cancer spurred the development and approval of drugs addressing specific steps of this pathway, including gonadotropin-releasing hormone (GnRH) analogues that centrally suppress androgen synthesis, the cytochrome P450 17A1 (CYP17A) inhibitor abiraterone acetate, which locally inhibits androgen synthesis in the testis, prostate and adrenal glands, and competitive antagonists, including enzalutamide, apalutamide and darolutamide, which inhibit AR function [15,16,17,18,19]. Unfortunately, therapy resistance often emerges, ultimately leading to metastatic CRPC (mCRPC) for which treatment with taxanes, poly (ADP-ribose) polymerase (PARP) inhibitors and alpha particle-emitting radiotherapy are used [15,20]. These treatments are merely indicated for a subset of patients and effective for only a limited time so that additional, more effective life-prolonging approaches are needed.

**Figure 1:**
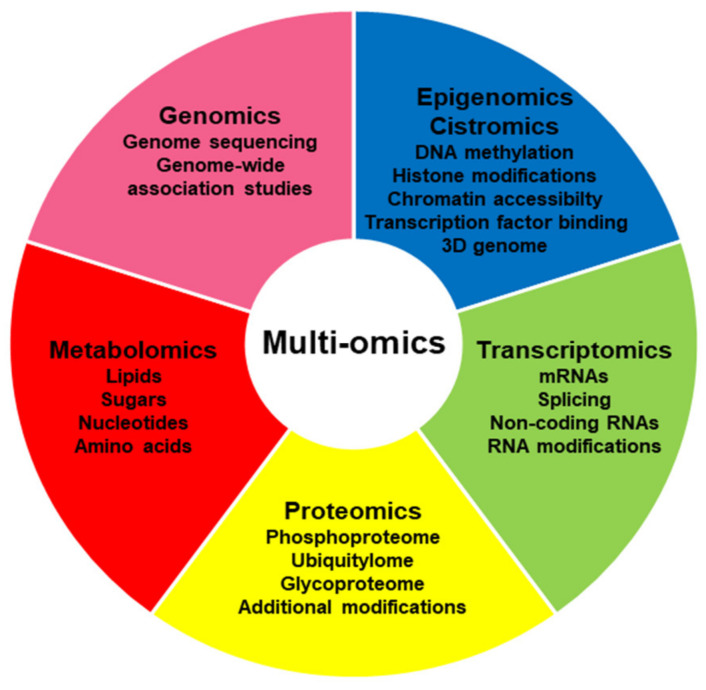
Overview of omics approaches.

A variety of preclinical models for prostate cancer, including cell lines, organoids, xenografts and genetically modified mouse models, as well as numerous patient-derived xenograft (PDX) models, are now available for evaluating the origins and therapy response of prostate cancer, but each of them can only provide limited answers [21]. Further, there is an increasing amount of data published from patients suffering from prostate cancer at an early or late stage and undergoing treatment. A diversity of omics-based platforms is now usable, including genomics, epigenomics, cistromics, transcriptomics, proteomics and metabolomics approaches, to examine the underlying process in detail and understand the various alterations taking place (Figure 1). Selected essential discoveries in each discipline will be summarized below and new, multi-omics procedures that integrate these findings to generate the bigger picture will be further discussed.

## 2. Genome Analysis

Functional genomics aims to generate and analyze genome data at a large scale. Whole-genome analysis of tumors is usually performed starting from biopsies of primary tumors or metastases from lymph node, bone, liver or soft tissue. Plasma DNA or circulating tumor cells obtained from liquid biopsies represent a further valuable source, which may bypass the problem of intra-patient variability and allow one to follow genomic variations along disease progression and treatment [22]. Importantly, the concordance between somatic modifications observed in liquid biopsies and matched tumors is substantial, 90.9% according to one study [23].

### 2.1. Genome Sequencing and Mutagenic Landscape of Early and Late Prostate Cancer

Prostate cancer has a comparatively low mutation rate and a highly variable number of gene copy number alterations [3]. The average mutation rate rises between primary and metastasis samples, from 1.36 to 2.93 per Mb according to one report [24], and is relatively low compared to other tumors [8,25,26]. Several comparative studies examining prostate cancer patients along disease progression have been published. Frequent modifications in the DNA damage repair and phosphatidylinositol 3-kinase (PI3K) pathways have been reported by several groups [24,27,28]. Mutations in the mitogen-activated protein kinase (MAPK) pathway and in the speckled-type POZ protein (*SPOP*) gene are also frequent events [24,27]. Putative driver genes with mutations in coding or non-coding regions have been further identified [29]. Importantly, many genomic alterations vary along tumor progression, as outlined below.

It is important to mention that most of the studies published concern a majority of patients of Caucasian origin, which limits their interpretation, considering the ethnic diversities described for prostate cancer [3,30,31,32,33]. For instance, a very recent publication points out differences in the rates of *PTEN* mutations and *AR* alterations in Black men, and in the rates of forkhead box protein A1 (*FOXA1*) mutations and zinc finger homeobox protein 3 (*ZFHX3*) alterations in Asian men, when compared to White men [34].

#### 2.1.1. Genome Sequencing of Primary Tumors

Comprehensive molecular analysis of primary tumors from a cohort of 333 patients defines seven molecular subgroups based on the presence of various *ETS* transcription factor fusions or mutations of *SPOP*, *FOXA1* and isocitrate dehydrogenase 1 (*IDH1*) [8]. These seven subgroups classify 74% of all primary prostate cancer cases. High heterogeneity is also observed following whole exome sequencing of 12 radical prostatectomy samples [35]. The most common alteration reported in primary prostate tumors is the fusion of the coding region for the *ETS*-related gene (*ERG*) or other *ETS* family members with an androgen-dependent promoter, usually from the transmembrane protease serine 2 (*TMPRSS2*) gene [3,8,24], leading to enhanced expression levels [36]. This fusion occurs early in prostate cancer development [37] and is associated with diagnosis and a more positive clinical outcome [38,39]. *SPOP* and *FOXA1* mutations also represent frequent early events [40,41]. *SPOP* inactivation leads to the stabilization of a number of proteins involved in prostate cancer, such as the AR, ERG, tripartite motif-containing 24 (TRIM24) and the bromodomain-containing proteins (BRD) BRD2, BRD3 and BRD4. Chromodomain helicase DNA-binding protein 1 (*CHD1*) and breast cancer 1 (*BRCA1*) are both involved in DNA repair and often altered early during the development of *ETS*-fusion-negative tumors [29]. Alterations described in primary prostate tumors that are potentially actionable in the clinic encompass DNA repair defects, epigenetic regulation changes and activation of the cell cycle, PI3K, Wnt and Ras signaling pathways [8,24].

#### 2.1.2. Genome Sequencing of Metastatic HSPC (mHSPC)

The examination of samples from primary tumors and metastases from 424 mHSPC patients shows enrichments in the Notch, cell cycle and epigenetic modifier pathways as most prominent in high-volume disease [42]. Numerous mutations in *PTEN* and *TP53*, and modifications in DNA repair genes and in the Wnt pathway were also disclosed [42,43]. A review of 11 studies comprising 1682 mHSPC patients shows changes in *TP53*, DNA damage repair and the Wnt pathway to be frequent [44]. A less favorable clinical outcome is observed in the case of alterations in *AR*, *Myc*, *TP53* or cell cycle signaling [44]. Dominant-negative *TP53* mutations are associated with a negative outcome and *SPOP* mutations with a favorable outcome in mHSPC patients [45].

#### 2.1.3. Genome Sequencing of mCRPC

*AR* gene amplification and enhancer hijacking are found in over half of mCRPC patients and this plays an important role in resistance to AR signaling inhibitors [24,26,35]. This leads to overexpression of full-length AR but also of several splice variants, including AR-V7 [46,47]. The emergence of AR mutations, essentially in the ligand-binding domain, followed by loss of inhibitory effects of AR antagonist medication, is observed in 10–30% of CRPC patients [48,49]. A generally greater mutational burden in *AR*, *TP53*, retinoblastoma protein (*RB1*) and *PI3K*/*AKT*, compared to matched hormone-naïve samples, is observed in mCRPC patients [28,43,50,51]. A study on 150 mCRPC patients defined *AR* and *TP53* alterations as the main modifications observed when compared to primary cancer [26]. Alterations in DNA damage response are often reported in mCRPC, mainly in the *ATM* and *ATR* serine/threonine kinase genes, and in the *BRCA* genes [27,52,53]. A recent investigation of circulating tumor DNA from 3129 mCRPC patients indicates an elevated 8.8% frequency of *BRCA1* and *BRCA2* mutations, more pronounced in the group with prior taxane therapy, and furthermore, a great concordance with data from tissues [54]. The frequent changes described in DNA repair pathways led to efforts towards developing PARP inhibitors to exploit this tumor liability, and two such compounds are now approved for prostate cancer cases with specific alterations [43]. In addition, mutations in the pioneer factor *FOXA1* and in the histone lysine N-methyltransferase 2C (*KMT2C*) genes are often observed at the CRPC stage [43]. A comprehensive investigation of 429 mCRPC patients led to the highlighting of *RB1* loss as the event most strongly linked to poor outcome [51]. A recent genome-wide study looked specifically at somatic mutations located in the 5′untranslated gene regions in five PDX models for mCRPC [55]. Many of the disclosed mutations are localized in regulatory elements representing DNA- and RNA-binding motifs and are associated with variations in gene expression and RNA translation [55].

Neuroendocrine differentiation leading to AR-independent growth is diagnosed more and more frequently, due to the widespread use of potent AR inhibitors [56]. Genetic hallmarks include the absence of *AR* enhancer gain, loss of *RB1* and the amplification of the *N-Myc* gene [57,58]. Another growing late-stage diagnosis is double-negative prostate cancer that expresses neither AR nor neuroendocrine markers [13]. Interestingly, no genetic alterations distinguish this cancer type from AR-positive carcinoma and neuroendocrine disease. Development of this treatment-induced late disease stage is likely driven by transcriptional regulation events.

### 2.2. Genome-Wide Association Studies

Nearly 270 genetic loci with single-nucleotide polymorphisms (SNPs) associated with prostate cancer risk have been determined by genome-wide association studies [59]. Another report describes 100 SNPs, which individually confer only a small added risk but altogether, explain one-third of the familial risk of prostate cancer [60]. Many relevant risk alleles are in non-coding cis-regulatory regions and affect gene transcription [59,61,62]. In many cases, this leads to the formation of altered regulatory complexes and to shifts in chromatin three-dimensional (3D) architecture [59,62]. Links between SNPs and therapy outcome were further evidenced [60,63]. Examples include germline polymorphisms detected in genes involved in androgen metabolism and that are coupled to response to androgen-deprivation therapy [64]. Sequencing of mitochondrial genomes originating from 384 localized prostate cancer patients led to the discovery of a number of single-nucleotide variants associated with aggressiveness [65].

## 3. Epigenome

Epigenetic regulatory mechanisms controlling gene expression principally include DNA methylation and histone post-translational modifications and are globally being evaluated by epigenomic studies. DNA methylation is mostly found at CpG dinucleotides and leads to gene silencing. Genome-wide investigation of the DNA methylome is generally achieved by whole genome bisulfite sequencing [66]. Post-translational histone modifications, such as acetylation or methylation, can enhance or inhibit gene expression, depending on the individual marks added and positions targeted, and on their combinations [67]. The global mapping of histone marks is essentially performed by antibody-based chromatin immunoprecipitation (ChIP) enrichment followed by next-generation DNA sequencing [68]. Non-coding RNAs, such as microRNAs (miRNAs) and long non-coding RNAs (lncRNAs), represent additional epigenetic, trans-acting players involved in the control of gene transcription. Their levels are monitored in total RNA or in enriched small RNA fractions by next-generation sequencing [69,70].

### 3.1. DNA Methylation

Several large studies underline the role of altered DNA methylation profiles in localized and metastatic prostate cancer [8,71,72,73]. Numerous differentially methylated loci were uncovered when comparing intermediate-risk prostate cancer with benign tissue [74]. The analysis of DNA methylation at precise positions has been proposed for risk stratification and treatment response prediction by different investigators [75,76,77,78]. A very recent study examined the DNA methylome of primary prostate tumors in detail and identified several subtypes, including one associated with poor prognosis [79]. Genes silenced by DNA methylation are commonly associated with DNA repair, cell cycle and cell adhesion, growth suppression and apoptosis [73]. A large survey of 100 metastatic biopsies revealed the existence of multiple sites with considerable DNA methylation and changes in methylation to occur during tumor progression, mainly at hotspots and putative regulatory regions [80]. Interestingly, numerous hypomethylated regions are present around the *AR* gene [80]. A distinct DNA methylation profile characteristic of treatment-emergent small-cell neuroendocrine cancer was found [80]. Radiation therapy has limited impact on overall DNA methylation in prostate cancer cells, with the exception of a few CpG sites [81].

### 3.2. Histone Modifications

Variations in histone marks, predominantly at the N-terminal tail, affect chromatin compaction and DNA accessibility [82]. Early immunohistochemistry and tissue microarray studies indicate that the combined patterns of histone H3 and H4 acetylation and dimethylation detected in low-grade prostate cancer are predictors of tumor recurrence [83]. H3K27 acetylation at enhancers bound by the AR, FOXA1 and homeobox protein HOXB13 are essential for enabling androgen-driven gene transcription [72,84,85]. Striking differences in histone H3K27 acetylation patterns are noticed between primary and metastatic prostate cancer samples [84,86]. Further, H3K9 di- and trimethylation as well as H3K4 monomethylation are reduced in prostate cancer, compared to normal tissue, but elevated in resistant tumors [86]. Monoubiquitylation of the histone H2A is modulated by androgen at numerous genes, leading to the expression regulation of several homeobox genes and control of cell growth [87].

### 3.3. Non-Coding RNAs

Competing roles of miRNAs and lncRNAs in regulating gene expression and an additional role of miRNAs in repressing translation in AR-independent prostate cancer and in NEPC have been reported [88]. There is also a direct impact of a number of non-coding RNAs on *AR* gene transcription, whereas the level of several of them is controlled by the AR [89,90,91]. Shifts in non-coding RNA abundance following therapy and an implication in resistance to AR antagonists or taxanes have been reported [92]. For instance, expression of the lncRNA HOTAIR is up-regulated by androgen deprivation, thus, leading to AR stabilization, and this promotes prostate cancer growth, invasion and metastasis [93,94]. Non-coding RNAs may serve as valuable biomarkers or even as novel therapeutic targets. Some tumor-suppressive miRNAs have been proposed as treatment options for prostate cancer, due to their involvement in cellular stemness and epithelial-to-mesenchymal transition (EMT) [95,96].

## 4. Cistrome

The genome-wide evaluation and interpretation of transcription factor binding is called cistromics. Epigenomic variations, such as DNA methylation and histone post-translational modifications, which also form part of the cistrome, were discussed in the previous paragraph. The technology of choice is usually ChIP, followed by microarray analysis or parallel DNA sequencing [68]. Chromatin accessibility assays allow one to delineate areas recognized by regulatory proteins [97,98]. Chromatin conformation capture analysis is performed to establish the 3D architecture of chromatin and to evaluate long-range interactions of DNA-bound proteins forming topologically associating domains (TADs), which represent important transcription regulation mechanisms [99,100]. Advances in ultra-high-throughput sequencing methods, single-cell studies and developments of dedicated bioinformatic pipelines have much expanded our understanding of the interconnections between chromatin organization and genome function in recent years.

Investigation of cell lines representative of normal tissue or prostate tumor by the Hi-C chromosome conformation capture method, which determines all possible pairwise interactions between genomic regions, led to the identification of 300 to 1000 TADs, including a group characteristic of cancer [101]. Cancer-specific TADs possess unique domain boundaries that retain binding of the transcriptional repressor CTCF and are enriched in histone H3K4 trimethylation [101]. A subsequent study performed at an increased resolution describes over a thousand TADs with altered sizes and epigenetic states between normal prostate and tumor cells [100]. The tumor-distinctive TADs are usually smaller in size and more transcriptionally active [100]. Enhancer-promoter loops were further studied and binding motifs for AR, FOXA1, ETS and grainyhead-like 2 (GRHL2) detected [100]. Comparison of cell lines modelling the individual stages of prostate cancer progression demonstrates the occurrence of adaptations in genome 3D architecture, mainly towards the opening up of chromatin, and adjustments in size and boundaries of TADs [102].

### 4.1. AR Cistrome

The AR impacts gene expression upon androgen stimulation by binding to multiple chromatin regions. Genome-wide studies show that it predominantly makes contact with distal cis-regulatory elements located remotely from transcription start sites [103,104,105]. Interactions between AR-activated enhancers and promoters leading to downstream gene activation have been studied in detail in prostate cancer cell lines [106]. Androgen-dependent AR interaction with enhancers is consistently reversed by additional treatment with the anti-androgen darolutamide [85]. This is paralleled by changes in binding by FOXA1 and BRD4, and in the levels of histone H3K27 acetylation and H3K4 monomethylation [85]. In addition, androgen-dependent AR connection with super enhancers, as defined by MED1 binding, and its reversion by darolutamide, have been reported [85]. A further study indicates that treatment with the AR antagonist enzalutamide alters the AR cistrome and leads to co-occupancy by an enhancer of zeste homolog 2 (EZH2), causing the cells to shift from the epithelial lineage [107]. Prostate cancer cells with resistance to enzalutamide display chromatin reprograming and modifications in AR and Myc binding compared to sensitive cells [108]. *CHD1* loss is selectively observed in prostate cancer and mutually exclusive with *PTEN* deletion, due to synthetic lethality [109]. This leads to redistribution of the AR cistrome and drives tumor formation in a murine model, associated with an up-regulation in oncogenic pathways [110]. The *SPOP* mutation leads to changes in the zones accessed by the androgen-stimulated AR in mouse prostate organoids [111]. AR occupancy is further altered by MED19 overexpression, consequently favoring androgen-independent growth in cooperation with ELK1 [112]. Interaction of SMARCA4 with AR-binding sites leads to the regulation of genes involved in cell adhesion and extracellular matrix organization [113]. ChIP-Seq analysis of prostate cancer cell lines revealed AR peaks in several genes involved in lipid synthesis [114].

Progression from normal prostate to the cancerous state is associated with AR cistrome rearrangement, thus, causing an extensive gene program switch from differentiation and growth suppression towards survival and proliferation [84,115,116,117,118]. In addition, numerous AR-binding regions not previously described in prostate cancer cell lines are found in CRPC tissue [119]. A survey of AR binding and H3K27 acetylation, H3K4 trimethylation and H3K27 trimethylation in 100 primary prostate cancer samples allowed researchers to define three major subgroups [120]. Comparison between healthy tissue, primary prostate tumor and CRPC shows the AR cistrome to vary significantly [84,115,121]. On the other hand, a comparative study of four metastases from one mCRPC patient reveals that most AR-interacting sites on the genome are shared, and they are additionally characterized by strong FOXA1 occupancy and histone H3K27 acetylation [122]. Mutations in AR-binding sites, as well as in ERG and FOXA1 sites, have been found in susceptibility SNP studies [123].

The splice variant AR-V7 appears in a substantial proportion of advanced CRPC cases and is associated with a reduced response to AR inhibitors [46,47]. It lacks the ligand-binding domain but has an intact DNA-binding domain and N-terminal region. It is constitutively active and detected in 75% of metastatic tumors, but very rarely in primary tumors [46]. It forms heterodimers with the AR and the two cistromes overlap [124,125,126], but additional, specific areas recognized by AR-V7 homodimers also exist [125,127,128]. AR-V7 and other splice variants are localized in the nucleus at open chromatin regions where preferential binding sites are present [129]. Chromatin recognition of AR-V7 is much dependent on HOXB13 [127,130]. Distinct AR-V7 cistromes were described in the 22RV1 and LNCaP95 cell lines, and colocalization with HOXB13 was evidenced [127]. Comparison of AR-V7 genome binding in tissues from three CRPC patients indicates a broad diversity, in line with the distinctive gene sets regulated [127]. AR-V7 furthermore connects with zinc finger X-chromosomal protein (ZFX) at unique attachment sites in gene regulatory parts [125].

### 4.2. FOXA1 and HOXB13 Cistromes

The enrichment of FOXA1 and HOXB13 cistromes at precise locations is observed in primary prostate tumors compared to normal adjacent tissue [131]. FOXA1 is essential for AR reprogramming towards a cancerous phenotype and its overexpression, together with that of HOXB13, leads to a shift in the AR cistrome in an immortalized prostate epithelial cell line towards a tumor-like phenotype [132]. *FOXA1* is mutated in 3–12% of primary and advanced prostate cancers, and several of these mutations alter the genome interaction and affect differentiation programs [120,133]. Simultaneous FOXA1 and AR binding is detected at androgen-controlled enhancers [85]. A recent publication reports the FOXA1 cistrome to be shifted in NEPC towards neuroendocrine-specific regulatory elements [134]. Homeobox protein HOXB13 prevents AR binding to cognate response elements but may also form heterodimers with the AR to stimulate downstream gene transcription [135]. Genomic binding of HOXB13 overlaps with AR-V7 but not with full-length AR [127].

### 4.3. ZFX and Sex-Determining Region Y High-Mobility Group-Box 2 (SOX2) Cistromes

ZFX is a DNA-binding factor of the krueppel family. It shows strong co-occupancy with AR-V7 and BRD4 at defined genomic areas, leading to activation of characteristic downstream targets [125]. Gene programs activated by the ZFX/AR-V7/BRD4 complex include cell cycle, autophagy and Wnt signaling [125]. The transcription factor SOX2 binds to DNA via its conserved high-mobility group box [136]. The *SOX2* and *SOX9* genes are overexpressed in advanced prostate cancer and *SOX9* is linked to decreased response to early treatment and to biochemical recurrence [137]. Cistrome analysis reveals that genomic sites bound by SOX2 in prostate cancer cells vary from the canonical ones, leading to oncogenic pathway activation and metabolic reprogramming [138].

### 4.4. ERG Cistrome

Association sites for ETS transcription factors, including ERG, are found in proximity to AR binding elements, but the overlap is only partial [103,139]. ERG expands AR attachment and transcriptional activity, thus, promoting tumor progression [140,141,142]. ERG phosphorylation does not affect genomic contact but leads to a loss of repressive activity in prostate cells [143,144].

### 4.5. N-Myc Cistrome

N-Myc interaction with chromatin is reprogrammed in NEPC, causing the silencing of genes of the epithelial lineage [145]. Interestingly, the N-Myc cistrome strongly overlaps with positions bound by FOXA1 and HOXB13. A synergy between N-Myc and *RB1* loss leading to highly metastatic prostate tumors was reported in a genetically engineered mouse model [57].

## 5. Transcriptome

Detailed transcriptomic profiles of cells or tissues are now mostly generated by next-generation sequencing techniques. They can focus on mRNAs but may include non-coding RNAs, such as miRNAs, lncRNAs or circular RNAs (circRNAs) [146,147,148,149]. RNA modifications, such as N6-methyladenosine methylation, are also being examined at a large scale due to their impact on stability and splicing [150]. The potential of gene expression data to provide additive value to traditional clinical parameters for disease progression prediction was shown several years ago in early microarray-based studies [151] and propelled the hunt for gene transcription signatures of disease recurrence and therapy response. Comparison of gene expression data from normal prostate, primary tumors and metastatic lymph nodes led to the identification of a 70-transcript signature, predictive for elevated risk of biochemical recurrence and metastasis [152]. Subtype categorizations centered on the activation of distinct pathways have been proposed and include the prostate cancer classification system based on 37 genes, and the PAM50 classification derived from a breast cancer algorithm and based on 50 genes [6,153]. More recently, a 26-gene transcriptional signature differentiating five mCRPC phenotypes was suggested, based on the expression of the *AR* or neuroendocrine genes [154].

### 5.1. Dysregulated Transcriptome—General Aspects

Gene signatures characteristic of the prostate cancer cell lines LNCaP and VCaP with pre-existing or treatment-induced resistance have been established using single-cell sequencing [108]. They highlight a reconfiguration of chromatin and reprogramming of transcription factor binding. An integrative survey of transcriptome data available for prostate cancer cell lines that respond or not to enzalutamide reveals many differentially regulated genes in both groups with pathways related to cell proliferation and protein degradation being selectively impacted in sensitive cell lines [155]. RNA-seq analysis of VCaP cells resistant to enzalutamide points out that the CXXC-type zinc finger protein 5 and its downstream target genes are up-regulated, and this is also seen in patient samples [156]. A detailed transcriptomic study of prostate cancer PDX models outlines the important role of the polycomb-repressive complex 2 (PRC2) and EZH2, of the G2-M checkpoints and of macrophage polarization in tumor progression [157]. Transcriptome analysis of three patient-derived prostate cancer organoids highlights important similarities with their matched primary tumor tissue, but not among the three models, and gene set enrichment analysis (GSEA) reveals an enrichment in pathways related to cell growth, metabolic activity and androgen-dependent gene regulation [158].

Transcriptome analysis of 87 samples from 23 patients with localized prostate cancer reveals that the heterogeneity among tumor foci from one patient is similar to the heterogeneity among different patients [159]. A comprehensive multi-cohort survey of publicly available transcriptomic data from normal, primary and metastatic prostate tissues highlights the role of *Myc*, alpha-methylacyl-CoA racemase (*AMACR*) and glutathione S-transferase P (*GSTP*) in tumor initiation, and of *AR*, *EZH2*, steroid 5a-reductase (*SRD5A*), tumor protein *TP63*, centromere protein A (*CENPA*) and PI3K catalytic subunit b (*PIK3CB*) in tumor progression [160]. In addition, several disease-stage-specific genes with prognostic significance were described. Transcriptome investigation of 101 CRPC metastases and integration with whole-genome data enabled the finding of essential tumorigenesis regulators, for instance, non-coding RNAs that enhance oncogene expression [161]. RNA sequencing of biopsies from 25 mCRPC patients responding or not to enzalutamide shows that gene sets linked to reduced AR activity and stemness are activated in non-responders [162]. Serum- or urine-based assays based on the expression of protein-coding mRNAs, mainly prostate-specific antigen (PSA), or non-coding RNAs for prediction of prostate cancer, are available [163].

Single-cell transcriptomic studies uncovered the existence of a luminal cell population in normal prostate cells with progenitor function and potentially involved in prostate cancer initiation [164]. Another single-cell RNA sequencing study identified a rare luminal subpopulation expressing stem-like genes and with the ability to regenerate following androgen ablation [165]. These findings may be essential for unraveling disease progression and metastatic dissemination. Furthermore, single-cell studies are key to understanding the local immune microenvironment. Although primary prostate cancer is considered to be a cold tumor with limited immune infiltration, single-cell RNA-seq studies reveal that there is an actionable immune environment in metastatic niches in the bone, as demonstrated in vivo in mouse studies [166].

### 5.2. AR-Regulated Transcriptome

Androgen treatment rapidly stimulates the transcription of hundreds to thousands of protein-encoding genes, but moreover, represses a number of genes, with a considerable overlap between different hormone-dependent prostate cancer cell lines [85,167,168,169]. A number of non-coding transcripts are also directly regulated by the AR [149]. An integrative examination of transcriptome data available for prostate cancer cell lines that respond or not to enzalutamide revealed many differentially regulated genes in both groups, with pathways related to cell proliferation and protein degradation being selectively impacted in sensitive cell lines [155]. A comparison between androgen-regulated genes in cultured LNCaP or in the corresponding xenograft shows the profiles to differ [119]. A 16 AR target gene signature that predicts recurrent prostate cancer and CRPC was, thus, proposed.

Early studies with clinical samples using microarray profiling indicate that androgen deprivation therapy reduces the expression of some, but not all, androgen-regulated genes in localized prostate cancer [170]. Evaluation of 20 androgen target genes in primary prostate cancer indicates that various patterns exist, depending on the presence of the *ETS* fusion or mutations in *SPOP* or *FOXA1* [8]. Examination of 429 mCRPC patients revealed high AR signaling, mostly in adenocarcinomas and low AR signaling in samples with neuroendocrine histologic features [51]. Bulk and single-cell transcriptomic evaluation of bone, liver and lymph node metastases of 14 mCRPC patients with resistance to enzalutamide shows the elevated transcription of genes linked to EMT and transforming growth factor β signaling [171]. Further, an overall up-regulation of AR isoforms, including AR-V7, is consistently seen. RNA-seq performed on tumor biopsies from CRPC patients with resistance to enzalutamide followed by GSEA allowed researchers to discover altered pathways and reduced AR function, as well as an activated stemness program in non-responders [162].

### 5.3. AR-V7 Transcriptome

A modified CWR22Rv1 cell line that expresses AR splice variants, but not the full-length form, retains the expression of AR target genes, but not their androgen dependency [172]. The AR splice variants control the levels of genes involved in DNA damage response, and ectopic AR-V7 expression stimulates the transcription of several genes involved in homologous recombination [172]. AR-V7 homodimerization and DNA interactions are needed for the mediation of DNA damage repair [173]. Unique gene transcription programs modulated by the AR or particular splice variants have been reported and there was limited overlap between the two prostate cancer cell lines expressing different AR variants [174]. A direct comparison of the transcriptional programs of AR-V7 and full-length AR reveals an increased expression of cell cycle genes [175]. RNA-seq investigation of the LNCaP95 prostate cancer cell line following AR-V7 silencing shows a preferentially repressive function of this splice variant, linked to partnering with members of the nuclear corepressor family and inhibition of histone H3K27 acetylation [126]. Several of the AR-V7 target gene products inhibit proliferation in CRPC cells [126]. Another study performed in LNCaP95 cells reveals the existence of 78 AR-V7 target genes, of which 4 are specific for AR-V7 [128]. Nucleoporin 210 (*NUP210*) and solute carrier family 3 member 2 (*SLC3A2*) were further analyzed and a strong reduction in cell proliferation observed after their expression knock-down [128]. Resistance to the anti-androgen enzalutamide correlates with high levels of full-length AR and AR-V7 and involves common and separate gene expression programs [176]. Comparison of the AR-V7 transcriptome with that of full-length AR indicates that a repressive function of the latter on genes involved in EMT is lost, which may impact tumor growth [169].

Examination of transcriptome data from CRPC samples shows a positive correlation between the abundance of RNA levels of AR splice variants, including AR-V7, and those of full-length AR [177]. The same study further shows that androgen deprivation prevents the negative feedback of AR on its gene transcription [177]. The expression ratio between AR-V7 and full-length AR increases upon the onset of the CRPC stage, compared to matched pre-treatment prostate tissues [178]. The AR-V7-regulated transcriptome varies a lot across CRPC patients but genes involved in tumor progression and additionally coregulated by HOXB13 are often detected [127]. Several genes up-regulated in patients with resistance to abiraterone and involved in tumor progression and poor survival are preferentially stimulated by AR splice variants [129].

### 5.4. Expression of Steroid Synthesis Genes

A further adaptation leading to resistance to anti-androgen therapy is the increase in lipid biosynthesis [179,180]. Androgens are mainly synthesized by a de-novo pathway starting with cholesterol, which is then converted after several steps to the main androgens testosterone and dihydrotestosterone. An alternative pathway involving progesterone has additionally been described. Individual enzymes involved in these pathways, including CYP17A1, 3b-hydroxysteroid dehydrogenases (HSD) 1 and 2,3-hydroxy-3-methylglutaryl coenzyme A reductase (HMGCR), SRD5A1, SRD5A2, aldo-keto reductase family 1 member C3 (AKR1C3) and steroid sulfatase, are elevated in CRPC, compared to primary tumors [181,182,183,184]. Distinct patterns of dysregulation and large differences between patients are observed, indicating several resistance mechanisms, leading to the stimulation of steroid synthesis. Conversely, a survey comparing samples from 1713 prostate cancer tissues and from 230 normal tissues found the expression of cholesterol synthesis genes to be reduced in tumors [185]. Importantly, there is a direct impact of androgens on the up-regulation of genes involved in lipid synthesis, for instance, sterol regulatory element-binding protein 1 (*SREBF1*), fatty acid synthase (*FAS*) and *AMACR* [186].

### 5.5. Expression of Non-Coding RNAs

An early work showed that prostate tumors have lower miRNA levels than benign prostate tissue, especially at the androgen-independent stage [187]. On the other hand, some miRNAs with oncogenic functions are overexpressed in prostate cancer [95]. Differences between the miRNA profiles in androgen-sensitive and -resistant cell lines, and in clinical samples, have been identified [188].

Non-coding RNAs found in extracellular vesicles are being evaluated as prostate cancer biomarkers [189]. Candidate miRNAs isolated from biological fluids have been proposed based on their up- or down-regulation, but no corresponding assay is currently being routinely used in prostate cancer patients [189]. Individual lncRNAs have been investigated for prostate cancer detection and a urine test for the measurement of prostate cancer antigen PCA3 is approved for clinical use [190]. Profiling of 31 normal adjacent and 143 prostate cancer samples highlighted distinct circRNAs with tumor-selective expression that could further be detected in extracellular-vesicle-enriched plasma samples and represent potential biomarkers [191].

## 6. Proteome

Proteomic techniques are used to estimate the complete set of proteins in cells, tissues or biofluids [192,193]. Early studies were mostly based on two-dimensional gel electrophoresis and only allowed to assess a limited number of proteins. Sensitive mass-spectrometry-based methods along with improved computational analysis are presently being used for high-throughout, comprehensive approaches and may involve protein labeling or not. Procedures for relative and absolute quantification have now been established. Current developments include reverse-phase protein microarrays, which warrant precise comparisons between healthy, diseased and treated tissues [194]. In addition, post-translational protein modifications, such as phosphorylation, ubiquitylation or glycosylation, are now likewise being investigated at a broad scale [195].

Proteome study of prostate cancer models grown in vitro or in vivo has been fruitful to gain insights into causal modifications related to treatment response and resistance [194]. Large-scale investigation of different androgen-dependent and -independent cell lines allowed researchers to find proteins linked to tumor progression and aggressiveness [196,197]. Proteomic analysis of the AR transcriptional complex in LNCaP cells led to the identification of numerous interactors essential for cell proliferation [198]. The impact of androgen and anti-androgens on the proteome was determined in prostate cancer cell lines and compared to transcriptomic data [199,200]. In several instances, a disconnect between the protein and transcript levels was noticed, but newer findings relate an overall better correlation [200]. A recent work focusing on rapid proteomic modifications following androgen application indicated five protein clusters to be involved in androgen signaling, and this was further validated in patient samples [201].

Concerning large-scale proteomic studies of clinical samples, a number of observations are now published [194]. The overlap among studies addressing primary tumors is, however, only partial, but proteins involved in metabolomic pathways, chiefly fatty acid synthesis, show consistent up-regulation. Lipid metabolism is also elevated in metastasized tumors, compared to primary ones [202]. The consistence between studies is altogether better in advanced prostate cancer where cell cycle and DNA damage response pathways are the most clearly altered [203]. In several cases, the correlation with genomic and transcriptomic data could be assessed, but was found to be limited, underlining the specific benefit of dedicated proteomic studies [203,204,205]. Additional interactome studies, mainly focusing on the AR, permitted a better understanding of the crosstalk with essential partners, such as FOXA1 and HOXB13 [105,113]. Additional interactomes important for prostate cancer growth involve N-Myc and ERG. Here, also, proteomic efforts allowed researchers to determine a number of interacting partners [145,206]. Another method focused on the spectral examination of blood samples from patients and led to the discovery of 404 proteins linked to prostate cancer [207]. In addition, a protein signature differentiating between pre- and post-radiotherapy-treated patients was evidenced [207]. A recent proteomic survey of normal and tumor prostate tissues from 22 patients led to the proposal of signatures involved in recurrence, with the most prominent network involving the YY1 transcription factor [208].

Changes in post-translational protein modifications have also been documented for prostate cancer. Phosphoproteome mapping of the LNCaP cell line reveals several AR cofactors and important transcription factors to undergo phosphorylation [209]. Examination of the LNCaP xenograft grown in intact or castrated mice outlines increased phosphorylation and activation of oncogenic pathways involving yes-associated protein 1 (YAP1) and p21-activated kinase 2 [210]. A dedicated phosphoproteomic study led to the discovery of definite kinase pathways involved in the formation of prostate cancer metastasis [211]. Irradiation of PC-3 cells rapidly induces changes in the phosphoproteome and increased AKT and MET phosphorylation is detected [212]. Numerous kinases and phosphatases interact with the AR and a shift in the phosphoproteome during tumor progression is observed in clinical samples [213]. Global phosphoproteomic studies of prostate cancer tissue samples permitted the finding of kinase targets and pathways related to progression, which may help for stratification of patients and selection of optimal medication [214].

Protein ubiquitylation is a marker for degradation but also for other cellular processes. Importantly, the ubiquitin ligase adaptor protein SPOP acts as a tumor suppressor and is mutated in about 11% of primary prostate cancers [215]. A global investigation of the ubiquitylome in a prostate epithelial cell model expressing cancer-associated SPOP alterations indicates that DEK and TRIM24 are essential substrates, and that DEK stabilization promotes cell invasion [216]. It regulates the stability of essential androgen pathway players, including the AR, steroid receptor coactivator 3 (SRC-3), TRIM24 and BRD4. TRIM24 and additional TRIM E3 ubiquitin ligases directly interact and control the function of the AR [217]. Several other players in the ubiquitin pathway are altered in prostate cancer [195] but an extensive investigation of how the global ubiquitylome is affected has not been reported.

Multiple variations in glycosylation patterns take place as prostate cancer progresses and the prostate is a major source of glycans [218]. PSA, prostate acid phosphatase and prostate-specific membrane antigen, which are encoded by androgen-regulated genes and represent key prostate cancer markers, undergo extensive N-glycosylation [218]. Aberrant N-glycosylation of numerous proteins, including extracellular matrix components, is observed during prostate cancer progression [219]. Importantly, the expression of several enzymes involved in glycosylation is under androgen control [220].

## 7. Metabolome

High-throughput technologies to identify and quantify diverse metabolites, such as amino acids, lipids, nucleotides and sugars in cells, tissues or biofluids, are available [221,222,223]. Analytical techniques are essentially based on nuclear magnetic resonance spectroscopy and mass spectrometry. Determining the causative impact of metabolomic variations on tumor progression, however, remains problematic, due to the impact on the cell redox state and role in DNA replication and transcription [224]. Further, a representative coverage of the metabolites remains a challenge with the current methodologies [225].

Major differences between the metabolomes of normal and cancerous prostate tissues have been observed, mainly in lipid and nucleotide metabolism, and also in the tricarboxylic acid (TCA) cycle, in polyamine synthesis and in the hexoamine biosynthesis pathway [226,227]. Elevated de-novo lipogenesis is a hallmark of prostate cancer [186] and different lipid forms are up-regulated in cell lines derived from prostate cancer metastases [228]. Androgens impact lipid metabolism at diverse tumor stages and there is a unique dependence of prostate cancer on fatty acids to drive progression [226,229,230,231,232]. Lipid profiles can now be mapped almost to single-cell level and several studies show that extensive and heterogenous rewiring takes place in prostate tumors [233]. Lipid metabolism is stimulated by androgens and plays a role in resistance to androgen deprivation therapy [186]. Comparison between prostate cancer tumor and normal adjacent tissue reveals high accumulation of cholesteryl esters [234]. Another, larger effort compared prostate cancer and benign prostate hyperplasia samples in 220 patients and also showed marked modifications in pathways linked to lipid metabolism [235]. Increased abundance of monounsaturated lipids and of elongated fatty-acid chains in phosphatidylinositol and phosphatidylserine lipids are noticed in prostate tumors, and importantly, phospholipid composition is altered in patients with response to AR inhibition [236]. Elevated baseline levels of circulating ceramides are associated with shorter overall survival in mCRPC patients [222].

Urine metabolomics represents a fast and sensitive strategy to determine diagnostic biomarkers for prostate cancer and potentially also prognostic response biomarkers. A metabolic signature for a diagnostic prediction has been proposed [237]. A number of metabolites, including several ones related to energy production, to amino acid metabolism and to the TCA cycle, are altered in urine, as reported in independent studies [223,238]. Some of these metabolic variations are similar in urine and prostate cancer tissue [221].

## 8. Integration of Omics Data

Single-omics approaches can only give a narrow view of the variations taking place at the individual cellular levels, which limits our comprehension of the causalities underlying a disease as complex as cancer. Further, each omics method has its own limitations related to the experimental setting, technical restrictions and bioinformatic analysis (Table 1). A broad multi-omics procedure will much increase our understanding of the interplay between separate information levels and lead towards a more comprehensive view of the alterations underlying the pathology (Figure 1). Achieving horizontal data integration across multiple studies and vertical data integration of various types of omics studies will be essential to generate the complete picture [239]. However, such an integration only aggravates the problem, linked to the high number of biological variables and the relatively low number of biological samples, making analysis a non-trivial issue. This necessitates the development of appropriate statistical analyses and informatic tools to integrate the accessible data. Algorithms mainly based on multivariate, similarity and network approaches, and on Bayesian consensus clustering, have been proposed [239,240,241]. Some methods are restrictive with regard to the types of data that can be used (e.g., some of the network methods that use known interactions between molecules) and others applicable in principle to any combination of datasets. An example for the latter is iCluster [242], which has been used in the Cancer Genome Atlas program and includes prostate data, for integrative clustering of patients based on multiple genomics data [8]. More detailed reviews of mathematical and algorithmic aspects of multi-omics data integration are available elsewhere [240,243].

Individual omics approaches all have respective strengths and weaknesses (Table 1) and have been essential in the identification of different key regulatory pathways and alterations occurring at different development stages of prostate cancer (Table 2). More recently, multi-omics approaches have significantly expanded our understanding of this disease [224,244,245]. A new multi-omics classification has been suggested [246] and pathways potentially amenable to pharmacological intervention, as well as crosstalk, for instance, between the epigenome and metabolic dysregulation, have been discovered [224]. Incorporation of genomic, transcriptomic and proteomic results from benign prostate hyperplasia and malignant prostate cancer uncovered commonly altered networks relevant for the tumor phenotype [247]. However, as mentioned above, the correlation of genomic, transcriptomic and proteomic data originating from prostate cancer patient samples is often limited. Proteomic changes taking place during prostate cancer progression are not reliably predicted by gene copy numbers, DNA methylation or transcript abundance, especially in advanced tumors [194]. Indeed, detailed proteomic evaluation of clinical samples already allowed researchers to unravel the role of pathways previously not predicted to be involved in prostate cancer by alternative omics methods [204]. A limited correlation between protein and RNA levels was also reported in two other studies, in localized prostate cancer samples and in distant metastases [203,205]. This may be due to improper storage of biological material or an insufficient number of samples analyzed [245].

## 9. Conclusions and Perspectives

Owing to impressive advances in high-throughput technologies, multi-omics approaches are now being intensively pursued for in-depth studies of individual cancer types and their response to therapeutic regimens [248,249,250,251]. Multiple levels of cellular perturbations can be investigated in detail in different tissues, tumor samples and in single cells. Spatial omics adds an additional degree of complexity by integrating data from the tumor microenvironment. Indeed, variabilities in gene expression profiles have already been observed in biopsies from selected areas of prostate tumors, including normal, PIN and cancer areas, using spatial transcriptomic approaches [252]. Taking into account the tumor ecosystem will much improve our future understanding of intercellular crosstalk and open new horizons in precision medicine.

Recent advances in computational methods for all these approaches will greatly help to unravel the interplay of the biological processes taking place and the mechanisms responsible for the switch from normal to cancerous phenotype. This will provide innovative opportunities in the early detection of the disease, risk stratification and decision on optimal treatment strategy. Many challenges, however, still remain due to heterogeneities among the omics technologies, the curse of data dimensionality with relatively few samples and a very large number of variables assessed, missing values in studies, and problems linked to the storage, annotation, interpretation and handling of substantial datasets.

Whole-genome sequencing data of over 2600 human cancers and nearly 1200 related transcriptomes are now available from the Pan-Cancer Atlas [253]. Additional portals with deposited omics data include the Genomic Data Commons Data Portal, the International Cancer Genome Consortium, the Cancer Genome Atlas, the Cancer Proteome Atlas, the Cancer Cell Line Encyclopedia, the cBioPortal and the Catalogue of Somatic Mutations in Cancer [239,249]. Clearly, limitations still exist for the use and optimal combination of all the availableinformation due to issues, such as complexity, heterogeneity, lack of harmonization and incompleteness, but advanced data integration strategies are being proposed to improve this [241,249].

Recent findings derived from integrated multi-omics approaches include predictors of clinical response, identification of novel potential drug targets, profiling of compounds and mechanisms leading to drug resistance in different tumor types, and discovery of processes underlying cell plasticity [254,255,256,257]. This is further complemented by recent advances in medical imagery, where the reliability of pathologic assessment has been much increased by refined molecular imaging techniques, and also by artificial intelligence and machine learning [224,244,258]. Altogether, this steadily broadening knowledge about the intricacy and heterogeneity of tumors throughout individual disease stages will advance informed precision medicine strategies for patients. Furthermore, it brings the additional hope that tailored prevention approaches will be available in the near future [259].

## Figures and Tables

**Table 1:** Strengths and weaknesses of omics approaches.

Approach	Strengths	Weaknesses
Genomics	Gives all sequence information on exons (whole exome sequencing) and additionally on introns, promoters, enhancers, intergenic regions, etc (whole genome sequencing) Identifies essential alterations	Prediction of final biological effect limited
Epigenomics	Gives information on potential regulation of genes	Dynamic nature and differences between cell types is often not reflected Correlation to gene expression may be limited
Cistromics	Describes genome architecture Gives information on gene regulation	Limited to specific binding factors or histone modifications analyzed Necessitates validated tools (e.g., high-grade selective antibodies) Correlation to gene expression may be limitedExpensive
Transcriptomics	Global expression analysis May detect all splice variants Sensitive, high dynamic range and quantitative Cell-specific transcriptomes can be resolved in single-cell experiments	Represents only an intermediate step Differences between organ- and cell-specific transcriptomes Correlation to protein levels not always linear
Proteomics	Addresses final regulation level Proteins are the main cellular effectors	Some proteins are difficult to separate High dynamic range of proteome makes detection difficult Absolute quantification necessitates labeling Individual experiments give only limited coverage Post-translation modifications may have strong impact on activity but can be difficult to analyze
Metabolomics	Close to phenotype Allows repetitive sampling of accessible biofluids	High diversity of metabolites of which only fraction is measured May be difficult to analyze and interpret

**Table 2:** Overview of main changes identified in prostate cancer using different omics approaches.

Approach	Major Findings Early Stage	Major Findings Late Stage
Genomics	Low mutation rate High copy number and structural aberrations *ETS-TMPRSS2* fusion *PTEN* deletion *SPOP* mutation *FOXA1* mutation	*AR* amplification and mutation *TP53* mutation *RB1* deletion *PIK3CA* amplification
Epigenomics	Identification of DNA hypermethylation subtype associated with disease recurrence	Hypomethylation around *AR* gene Changes in H3K27 acetylation Changes in H3K9 di- and trimethylation Upregulation of lncRNA HOTAIR
Cistromics	AR cistrome rearrangement ETS factor-driven AR cistrome reprogramming Enrichment of FOXA1 and HOXB13 cistromes	Novel AR-V7 cistrome Changes in ZFX and SOX2 cistromes
Transcriptomics	Identification of subtypes predictive of recurrence and metastasis Changes linked to *ETS* fusion Changes linked to *SPOP* or *FOXA1* mutation	Emergence of AR-V7 and other AR splice variants Expression upregulation of steroid synthesis genes
Proteomics	Ubiquitylome changes linked to *SPOP* mutation	Abundance of cell cycle and DNA damage response pathway proteins Role of FOXA1 and HOXB13 interactomes
Metabolomics	Changes in lipid and nucleotide metabolism Changes in TCA cycle, polyamine synthesis and hexoamine biosynthesis pathway	

## Data Availability

Only previously published data are discussed.
